# Pure sensory chronic inflammatory polyneuropathy: rapid deterioration after steroid treatment

**DOI:** 10.1186/s12883-015-0291-7

**Published:** 2015-03-11

**Authors:** Elisabeth Chroni, Dimitra Veltsista, Evangelia Gavanozi, Tavitha Vlachou, Panagiotis Polychronopoulos, Panagiotis Papathanasopoulos

**Affiliations:** Department of Neurology, School of Medicine, University of Patras, Patras, Greece; Department of Neurology, University Hospital of Patras, Rion, 26504 Greece

**Keywords:** Ataxic neuropathy, Chronic inflammatory demyelinating polyneuropathy, Sensory neuropathy, Steroid treatment

## Abstract

**Background:**

Chronic inflammatory demyelinating polyneuropathy (CIDP) as a pure sensory variant is rarely encountered. Therefore the best treatment option is hard to define.

**Case presentations:**

We reported two middle-aged patients of Caucasian origin, one female and one male, who over a period of several months presented limbs and gait ataxia. Clinical and neurophysiological examination revealed only sensory abnormalities. A diagnosis of atypical CIDP was suggested, considering the elevated CSF protein level and the presence of anti-gangliosides antibodies. Ten and 15 days respectively after initiation of prednisolone treatment both patients experienced exacerbation of sensory symptoms and emerging of muscle weakness. Steroids were then substituted by rituximab in the first patient and intravenous immunoglobulin in the second patient resulting in gradual decrement of symptoms and signs. Two-year follow-up showed no further deterioration.

**Conclusion:**

Caution should be exercised when treating cases of pure sensory polyneuropathy with high dose steroids since an unfavorable outcome is possible.

## Background

Pure sensory neuropathy is an uncommon condition which in most cases is considered idiopathic and categorized under the range of chronic axonal neuropathy or cryptogenic polyneuropathy [1,2]. Fewer cases of sensory polyneuropathy have been identified as an entity within the spectrum of chronic inflammatory demyelinating polyneuropathy (CIDP) [3,4]. The latter cases have at least subclinical involvement of motor components and thus fulfill the diagnostic criteria for CIDP [5,6]. In a recent study, 22 patients with responding to immunotherapy sensory polyneuropathy but without clinical or electrophysiological criteria for motor involvement were considered to have a pure sensory variant of CIDP [7].

The recognition of an auto-immune nature of the sensory neuropathy justifies treatment attempts with 1st line medication for CIDP. However, due to the relatively small number of cases reported in the literature the response to treatment is not well known. It is possible that as in other atypical forms of CIDP one drug may be preferable to another. We herein report on two patients with pure sensory polyneuropathy who experienced rapid deterioration following steroid administration.

## Case presentation

### History and clinical presentation

#### First patient

A 58 year old Caucasian female presented with a 12 month history of progressively worsening numbness in her hands and feet and impaired balance which caused infrequent falls. She was previously healthy, denied alcohol or chemical agents’ consumption and had a family history of bowel cancer. Neurological examination revealed diminished pin-prick and touch sensation in a stocking-glove distribution, severely reduced vibration sense to the knee level and impaired joint position sense at the great toe bilaterally. She had absent all deep tendon reflexes, but normal muscle strength throughout, intact cranial nerves and flexor plantar responses. The patient’s stance and gait was wide-based, tandem gait was impaired, Romberg’s sign positive and her score on the Overall Neuropathy Limitations Scale (ONLS) [8] was 3 (1 on the arm scale).

#### Second patient

A ship captain, 55 year old Caucasian male, presented with an 18 month history of symmetrical and progressive numbness and paresthesia in his feet, which over the last 6 months spread proximally and affected his hands as well. He had a personal and family history of coronary heart disease. He did not smoke, drink alcohol, use drugs or dietary supplements. On neurological examination there was reduced touch and pricking pain sensation distally up to the wrist and ankle level, suppressed vibration sense at the great toe bilaterally and generalized areflexia. Cranial nerves and muscle strength examination showed no deficits. Walk was unsteady, tandem gait was performed with difficulty, Romberg’s sign positive and his ONLS score was 1 (on the leg scale).

### Sensory assessment testing

To depicture the extension and severity of the sensory deficit we chose to present the findings by means of an extended version of Neuropathy Impairment Score–more specifically the sensation part- (NISsen) [9,10] in order to assess four sensory modalities: touch-pressure, pricking pain, vibration and joint position at the index finger, wrist, elbow, great toe, ankle and knee level bilaterally. Normal was marked as 0 score, decreased function as 1 and absent function as 2 score. In particular for vibration sense a Rydel-Seiffer graduated tuning fork was used with a scale imprinted on the weights from 0 minimum to 8 maximum. Quantitative vibration estimations up to 5 on the tuning fork scale were considered mildly decreased sense and were graded as 1, whereas estimations lower than 5 on the 0–8 scale were graded as 2. According to the above scoring system, the lowest limit on the total of all scales was 96. At presentation the first patient had a total score of 30 and the second patient 18.

### Neurophysiological examination

The patients underwent the following electrophysiological tests: a. motor conduction and F waves study of the ulnar, median, fibular and tibial nerves bilaterally using bipolar surface recording and stimulation electrodes; the compound muscle action potential (CMAP) was obtained from the abductor digiti minimi (ADM), abductor pollicis brevis (APB), extensor digitorum brevis (EDB) and abductor hallucis muscle respectively; b. magnetic stimulation at Erb’s point and C7 cervical root in order to elicit CMAP from ADM, APB and at the popliteal fossa and L5 lumbar root level to elicit CMAP from EDB muscle bilaterally; c. sensory conduction of the ulnar and median nerves (orthodromic technique, digital ring stimulating electrodes) and the radial and sural nerves bilaterally (antidromic technique, bipolar surface stimulating and recording electrodes); d. needle electromyographic sampling of biceps brachii, 1st dorsal interosseous, vastus lateralis and tibialis anterior muscles unilaterally; e. somatosensory evoked potentials (SSEPs) in the first patient only. Representative neurophysiological measurements are showed in Table 1. All parameters of motor nerve conduction were within normal limits except for the F wave maximum latency which was slightly prolonged in the second patient. Proximal conduction abnormalities i.e. motor conduction block, defined as >50% CMAP amplitude and area drop in proximal compared to distal stimulation, or temporal dispersion, defined as proximal duration increased > 30%, were not detected by magnetic stimulation. Sensory nerve action potentials (SNAPs) had reduced amplitude or were absent in the upper limbs, the sural nerve potentials were comparatively less affected and the sensory velocity normal (Table 1) There were no signs of denervation in neither of the patients. Briefly, the findings, which were similar in both patients, were compatible with sensory component disturbance that can be attributed to sensory neuropathy or neuronopathy. SSEPs to median nerve stimulation showed ill-formed responses with prolonged latency and low amplitude at the cortical level (N20 right/left: 26.5 ms/24.3 ms) and non-reproducible potentials at Erb’s point bilaterally; tibial nerve stimulation showed absent lumbar and cortical responses bilaterally.

**Table 1:** **Electrophysiological results in two patients with clinically pure sensory deficits**

*Motor conduction*						
	*Ulnar nerve*			*Fibular nerve*		
	Pt. 1	Pt. 2	Normal limit	Pt. 1	Pt. 2	Normal limit
Distal latency (ms)	2.8	3.1	<3.5	3.7	4.8	<5.0
Amplitude (mV)#	8.3/8.0	6.8/6.4	>5.0	3.9/3.2	3.4/3.1	>2.0
Duration (ms)#	4.6/5.3	5.1/5.7	<6.7§	4.8/5.4	6.3/6.5	<7.6§
Velocity (m/s)	60	50	≥50	46	39	≥41
F wave lat. min/max (ms)	31/35	32/37	<32/35*	50/61	55/69	<55/64*
F wave persistence (%)	90	80	>70	70	50	>20
*Sensory conduction*						
	*Median nerve*			*Ulnar nerve*		
	Pt. 1	Pt. 2	Normal limit	Pt. 1	Pt. 2	Normal limit
Amplitude (μV)	2.0	Absent	>10	1.7	Absent	>7
Velocity (m/s)	48	-	≥50	56	-	≥45
	*Radial nerve*			*Sural nerve*		
	Pat1	Pat2	Normal limit	Pat 1	Pat 2	Normal limit
Amplitude (μV)	5.9	4.5	>15	14	3.4	>7
Velocity (m/s)	63	58	≥55	62	43	≥45

#Measurements following stimulation at the wrist/above elbow and at the ankle/above knee.

*Calculated from samples of 20 F waves for the patients’ age and height; §duration limits were adopted from those published by EFNS/PNS [11].

### Laboratory evaluation

In both cases, the following blood tests were normal or negative: complete blood count, erythrocyte sedimentation rate, blood glucose, electrolytes, renal and liver function tests, thyroid function tests, serum protein electrophoresis and serum immunofixation, serum vitamin B12, vitamin E (70 and 76 μg/ml in the 1st and 2nd patient respectively), C-reactive protein, antinuclear antibodies, anti-SSA & anti-SSB, c-ANCA & p-ANCA, RPR, HIV, HTLV and paraneoplastic antibody screening, which included antibodies against Hu, Yo (1st patient only), amphiphysin, SOX1 and CV2/CRMP5. Testing for anti-ganglioside antibodies was negative for anti-MAG (IgM class), GM1, GM2, GQ1b, GD1b, GD3 and sulfatide in both subjects, strongly-positive but without specific isotype detection for anti-GD1a and GT1b in the first patient and for anti-GT1a in the second patient. Cerebrospinal fluid (CSF) examination showed a protein level of 0.65 g/L (normal <0.45 g/L) with 3 white blood cells in the first case and 1.53 g/L with 11 white blood cells in the second case supporting an albuminocytologic dissociation. In both patients Schirmer-I test and Rose-Bengal score were negative; chest and abdomen computed tomography scans, mammography (1st patient) and colonoscopy showed no signs of malignancy, whereas magnetic resonance imaging scans of the brain, cervical and lumbar spine were negative.

### Treatment and follow-up

**Figure 1:**
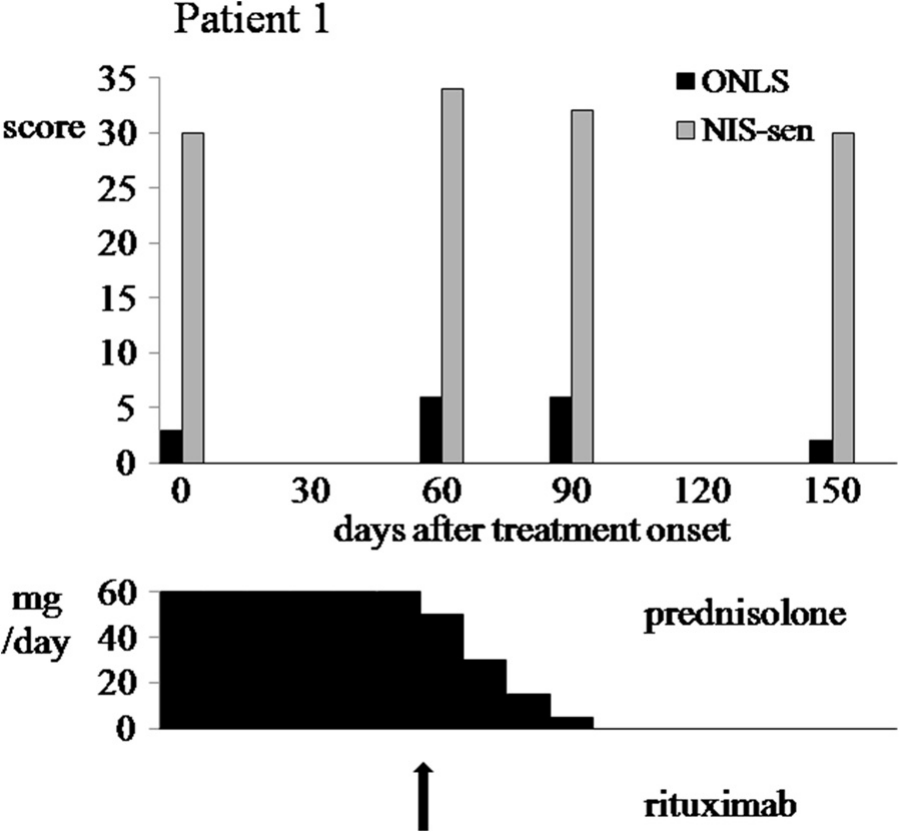
**Clinical assessment scores of a 58 year old woman are plotted against treatment regime at different time-points.**

The first patient was initially treated with prednisolone (60 mg p.o. daily) but within 10 days she reported increase of unsteadiness and numbness which also involved the trigeminal nerve region. Nevertheless, according to the instructions she completed a two-month period of steroid treatment. At that time her sensory deficits enhanced, she manifested weakness of quadriceps and wrist extensor muscles (expanded MRC score reduced from 70 to 66) and became unable to walk without bilateral support. She scored 6 on the ONLS (2 on the arm scale). Prednisolone was tapered off and intravenous immunoglobulin (IVIg) therapy was started at a dose of 0.4 mg/kg/day. The second day of infusion the patient manifested severe headache, nausea, fever, nuchal rigidity and confusion. A second CSF analysis showed neutrophilic pleocytosis (700 white blood cells/mL, normal glucose and 0.93 g/L protein). Following thorough laboratory investigation a diagnosis of aseptic meningitis was made and IVIg treatment was discontinued. A course of rituximab (i.v. infusions at a dosage of 375 mg/m^2^ weekly for 4 weeks) was tried as a second-line treatment according to recent literature [12]. Three months following steroid withdrawal the patient’s clinical status returned to pre-steroid level (Figure 1). Two years later, following another two rituximab infusions, the patient was able to walk unaided and reported only minimal distal numbness of her hands and feet.

**Figure 2:**
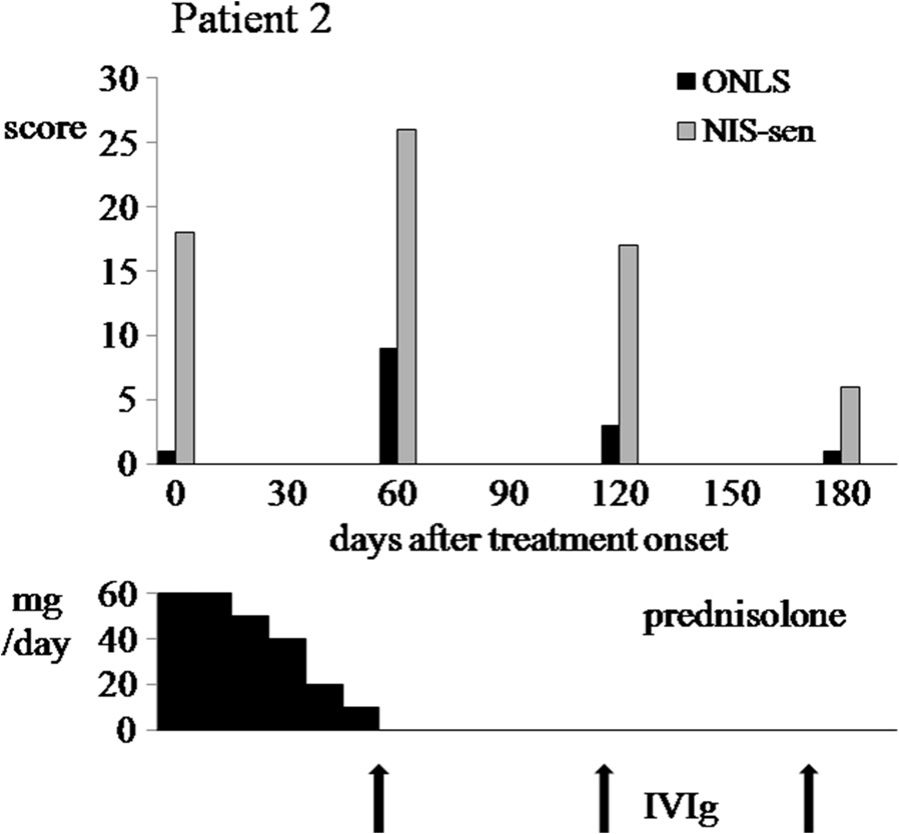
**Clinical assessment scores of a 55 year old man are plotted against treatment regime at different time-points.**

The second patient was started on oral prednisolone (60 mg daily) but from day 15 he experienced exacerbation of ataxia and spreading of the numbness. Steroid tapering was scheduled 20 days from treatment onset since the patient developed diplopia, numbness in the face, inability to perform fine hand’s movements, stand or walk 1 meter (expanded MRC score 64; ONLS arm grade 4, leg grade score 5). Within the next month the patient’s condition improved significantly; his cranial nerve deficits and muscle weakness disappeared and he was able to walk again with support for a few meters. Monthly infusions of IVIg at an initial dose of 0.4 g/kg daily for 5 days and maintenance dose of 0.5 g/kg for 2 days) had a beneficial but short-lived effect on this patient, requiring monthly treatment repetitions (Figure 2). Over the next two years the patient’s condition was further improved by IVIg infusions every 4–6 months and he was able to return to his work.

## Discussion

The disease characteristics, shared by the two patients were: i. pure sensory deficits; ii. a chronic, slowly progressive course, over the duration of several months; iii. sensory ataxia that predominated clinically, affecting both the upper and lower limbs; iv. early areflexia throughout; v. raised protein levels without cells in the CSF; vi. low amplitude of (SNAPs), especially the “abnormal ulnar-radial/normal sural” pattern, along with the absence of any motor nerve conduction abnormalities. Two additional characteristics were added shortly (10–15 days) after administration of prednisolone: vii. cranial nerve involvement and viii. muscle weakness. Sensory ataxia could theoretically be due to dorsal root ganglionopathy [4,13]. According to the American-European Consensus Group, a diagnosis of Sjögren’s syndrome was not established in our patients, since neither of them fulfilled the requirement for the presence of 4 out of 6 criteria [14]. More specifically, both patients had no ocular or oral symptoms, ocular signs and anti-SSA & anti-SSB antibodies. A paraneoplastic syndrome, which cannot be entirely ruled out, is highly unlikely given the above disease’s characteristics iv-vii. A diagnosis of chronic axonal polyneuropathy was also considered. However, the predominance of ataxia and areflexia in the absence of neuropathic pain, the presence of anti-gangliosides antibodies and the increased CSF protein level implying an intrathecal process, made the diagnosis of sensory polyradiculo-neuropathy more suitable [1]. According to the current criteria set by the European Federation of Neurological Societies/Peripheral Nerve Society our cases should be considered as atypical CIDP [11]. Indeed, the authors of a retrospective study described patients with the above clinical features of a chronic sensory polyneuropathy and concluded that they all had sensory CIDP [7]. We acknowledged the omission of spinal roots/ plexus MRI and nerve biopsy in our patients, which may not be necessary examinations for the diagnosis, but they would have further supported our view.

Immune-mediated sensory polyradiculo-neuropathies have long been recognized as a specific type of CIDP. The majority of patients with only sensory symptoms are likely to develop subclinical motor conduction abnormalities and thus become compatible with CIDP [3,5]. However, some patients have normal nerve conduction measurements but abnormal somatosensory evoked potentials [4] and others, as our patients, have sensory conduction abnormalities indicative of chronic axonal polyneuropathy [15,16], thus failing to meet the electrodiagnostic criteria for definite CIDP [11]. Nevertheless, it was decided the patients receive treatment since their neurological condition had a prolonged negative impact upon daily living activities. According to guidelines for CIDP sufferers, including patients with pure sensory manifestations [5,7,11,17,18] and availability of medication at the time prednisolone was our first choice.

Initiation of steroids was followed by severe worsening of disability which in both cases was completely reversible after steroid substitution with alternative medication. The possibility of incidental deterioration, irrespective of treatment, was unlikely since prior to the administration of steroids both patients had a slowly progressive course over several months which allowed them to be independent and continue to work, while within a few days of treatment onset ataxia increased to the extent that they were unable to walk unaided, developing motor impairment for the first time. The re-initiation of steroids in our patients could possibly have supported our hypothesis on their negative effect, yet it was not attempted for ethical reasons, whilst both patients did not wish for us to do so. A single case of pure sensory CIDP where severe deterioration was noted within days of steroid administration has recently been reported in the literature [19]. In a series of 13 patients with chronic sensory ataxia and positive anti-GD1b antibodies IVIg administration was successful in 9 patients, whereas steroid treatment resulted in a moderate improvement in only 1 out of 4 patients [20]. It could be assumed that steroids alter the balance of lymphocyte subpopulation in favor of B cells, thus increasing the circulating autoantibodies [21]. An unexpected motor deterioration after plasma exchange has been reported in two patients with sensory CIDP and this effect was attributed to imbalance between pathogenic and protective autoantibodies [22]. Clinical trials have demonstrated worsening in typical and atypical CIDP cases under steroid treatment [23,24]. Specifically for pure motor CIDP recommendations against the use of steroids have been given [11,25]. In analogy, the unfavorable response to steroid treatment in the present study implies that prednisolone may be harmful in some cases of sensory CIDP.

## Conclusion

We suggested that caution should be exercised when treating cases of pure sensory polyneuropathy with high dose steroids since an unfavorable outcome is possible.

## Consent

Written informed consent was obtained from both patients for publication of this Case report. A copy of the written consent is available for review by the Editor of this journal.

## Abbreviations

CIDP: Chronic inflammatory demyelinating polyneuropathy
ONLS: Overall Neuropathy Limitations Scale
NISsen: Neuropathy Impairment Score - the sensation part-
SNAPs: Sensory nerve action potentials
IVIg: Intravenous immunoglobulin
